# Ethnoveterinary Study of Medicinal Plants in a Tribal Society of Sulaiman Range

**DOI:** 10.1155/2014/127526

**Published:** 2014-10-21

**Authors:** Akash Tariq, Sakina Mussarat, Muhammad Adnan, Naser M. AbdElsalam, Riaz Ullah, Abdul Latif Khan

**Affiliations:** ^1^Department of Botany, Kohat University of Science and Technology, Kohat 26000, Pakistan; ^2^Riyadh Community College, King Saud University, Riyadh 11437, Saudi Arabia; ^3^Department of Chemistry, Government College Ara Khel, FR Kohat 26000, Pakistan; ^4^Department of Biological Sciences and Chemistry, University of Nizwa, 616 Nizwa, Oman

## Abstract

The aims of the present study were (i) to document ethnoveterinary plants and their formulation techniques in an unexplored region of Pakistan and (ii) to select candidate medicinal plants with high consensus factor and fidelity value for further *in vitro* investigation. A total of 60 informants were interviewed using semistructured questionnaire. A total of 41 plants belonging to 30 families were used to treat livestock ailments in study area. Mostly leaves (47%) were used in recipes formulation mostly in the form of decoction. Gastrointestinal infections were found more common and majority of the plants were used against cow (31) and buffaloes (24) ailments. Recovery time of majority of the recipes was three to four days. Informant consensus factor (Fic) results have shown a high degree of consensus for gastrointestinal, respiratory, and reproductive (0.95 each) ailments. Fidelity level (FL) results showed that *Asparagus gracilis* ranked first with FL value 93% followed by *Rumex hastatus* ranked second (91%) and *Tinospora cordifolia* ranked third (90%). Aged farmers and nomads had more traditional knowledge as compared to younger ones. Plants with high Fic and FL values could be further investigated *in vitro* for the search of some novel bioactive compounds and young generation should be educated regarding ethnoveterinary practices.

## 1. Introduction

Traditional veterinary medicine was experienced as early as 1800 B.C. at the time of King Hammurabi of Babylon who devised laws on veterinary fees and charged for treating animals [1]. Many ethnoveterinary medicines were neglected due to the development of initial western drugs. Ethnoveterinary practices have gained tremendous importance for the last decade due to the discovery of some effective ethnoveterinary products [2]. Traditional veterinary medicines provide a cheap therapy and easy accessibility in comparison with western drugs [3].

Ethnoveterinary practices are more common in developing countries due to different socioeconomic factors [4]. Pakistan is an agriculture country and almost 80% of its population is dependent on agriculture and livestock. Pakistan is the world's 5th largest milk producing country due to its high dependency on agriculture and livestock [5]. Resource-poor farmers of Pakistan greatly rely on traditional medicine due to their limited access to modern prevention health practices and particularly lack of modern health facilities in their areas [6]. Despite the fact that traditional knowledge is very much important for the livestock health and productivity, the documentation of this knowledge is very much neglected in majority of the remote areas of Pakistan. Livestock farmers all over Pakistan can draw on over 4000 years of knowledge and experience [7]. This traditional knowledge has been passed orally from generation to generation but it may be extinct or may be endangered due to the current rapid socioeconomic, environmental, and technological changes [8]. Therefore, the documentation of such knowledge is very crucial before its extinction for future developments.

The present research study was therefore designed to document detailed ethnoveterinary knowledge of an unexplored region of Pakistan. The present study was designed with the objectives (i) to document ethnoveterinary plants of the Kohat region, (ii) to document the detailed formulation techniques of the reported ethnoveterinary plants, and (iii) to select candidate medicinal plants with high consensus factor and fidelity value for further* in vitro* investigation. The present study would provide baseline information to phytochemists, pharmacologists, and conservationists for further future research studies. This work would also make a great contribution to the conservation of this valuable knowledge.

## 2. Methodology

### 2.1. Study Area

**Figure 1:**
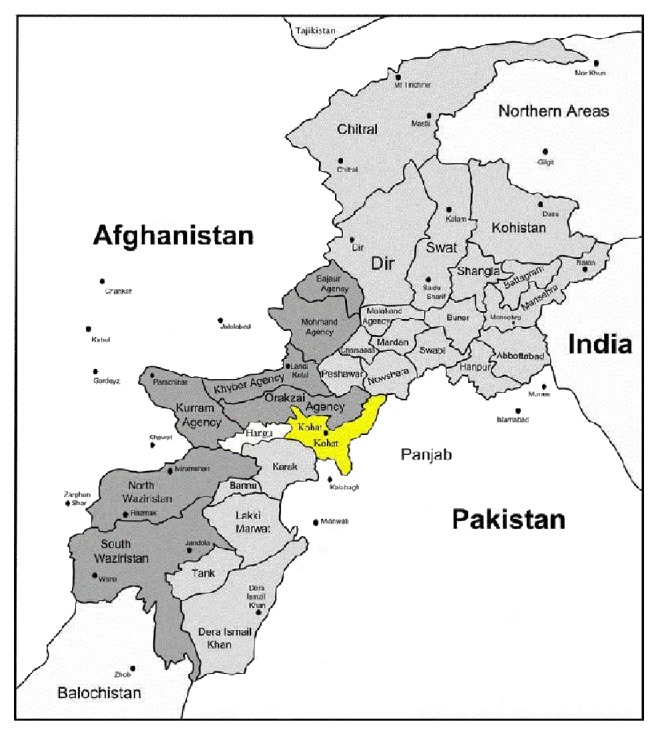
Study area map.

Kohat is a medium sized town in Khyber Pakhtunkhwa of Pakistan. It is located at 33°35′13 N, 71°26′29 E, with an altitude of 489 m, and is the capital of the Kohat District (Figure 1). Summer temperatures usually shoot above 50°C and winters are mild [9]. The dominant vegetation of the study area is* Zizyphus* species,* Acacia* species, and other xerophytes plants. The area is rural in nature and inhabitants are very much dependent on livestock for agricultural, economic, and food purposes. Locals of the region use a variety of medicinal plants for the treatment of livestock ailments due to expensive veterinary drugs.

### 2.2. Sampling and Data Collection

Field work was done from January to May 2014. Initially, local administrative officers and representative (*Malik*) of the study area were visited, who provided information on key resource persons in the field of ethnoveterinary medicinal plants. They suggested 60 informants having strong traditional knowledge regarding livestock treatment. Out of 60 informants 45 were farmers and 15 were nomadic people. A brief group discussion was held with the informants prior to data collection for explaining to them the main theme of the present study and to get their consent for the publication of their traditional knowledge. This was done in order to acknowledge informants' cooperation in preserving the traditional knowledge of the study area and build their confidence for providing reliable information. Each informant was separately interviewed in their local languages. Semistructured questionnaires were designed addressing detailed ethnoveterinary information (*Hindko*). Informants were asked about the number of plants they use to treat their livestock, which part of plant used, recipe formulation, recovery, and other essential questions.

### 2.3. Data Organization

Data collected from informants was organized using Microsoft Excel 2007 and Microsoft Word 2007. Plant habit was categorized into four classes, that is, herb, shrub, tree, and climbers. Plant parts were classified into leaves, stem, root, stem, whole plant, seeds, and fruit. Medicinal plants uses were categorized into 8 major categories, that is, gastrointestinal, dermatological, respiratory, reproductive, wound healing, antipyretic, parasitic, and general body tonic. Recipes were classified into different groups, that is, decoction, powder, crushed, juice, paste, poultice, infusion, and concoction. Route of administration was divided into 3 categories, that is, oral, dermal, and nasal.

### 2.4. Data Analysis

Informant consensus and fidelity level were used to verify the importance of medicinal plants.

### 2.5. Informant Consensus (Fic)

Fic on the reported cures of a given group of ailments was calculated as an informant consensus factor. Fic within a community designates the widely used plants and thus helps in the selection of plants for phytochemical and pharmacological studies [10]. Reported veterinary problems were grouped into 8 major ailments. Fic values are high when one or few plants are reported by the large number of respondents to treat a specific ailment, while low Fic values give an indication that informants do not agree over which plant to use [11, 12].

The Fic can be calculated using the formula as follows:

(1) $$\begin{array}{c} \text{Fic}=\frac{\text{nur}-\text{nt}}{\text{nur}-1}, \end{array}$$

where Fic = Informants consensus factor, nur = number of used citations in each category, and nt = number of species used.

### 2.6. Fidelity Level (FL)

FL is useful for recognizing the most preferred plants used for curing certain ailments by the respondents. Highly preferred plants have always greater FL values than those that are less preferred. FL is always calculated in terms of percentage of informants claiming the use of a certain plant species for the same major purpose. The main purpose of FL is to calculate the importance of plant species for a specific purpose. Prior to the calculation of FL values all of the ailments that were reported are grouped into major classes [10]. FL value was estimated using the formula FL = Ip/Iu × 100, where Ip is the number of respondents who reported the utilization of medicinal plants for a specific main ailment and Iu is the total number of respondents who mentioned the same plant for any ailment [13]. It is assumed that those medicinal plants which are plants used in some recurring manner for the same disease category are more likely to be biologically active [14].

### 2.7. Collection and Preservation of the Reported Medicinal Plants

Field trips were made with local informants for the collection of the reported medicinal plants. Collected medicinal plants were brought to the laboratory of Kohat University of Science and Technology (KUST), Kohat, Pakistan, for further processing. Specimen identification and confirmation were undertaken by using Flora of Pakistan and taxonomic experts. Plants were dried and pressed on herbarium sheets and deposited at the Herbarium of Department of Botany, KUST, Kohat, Pakistan.

## 3. Results

**Figure 2:**
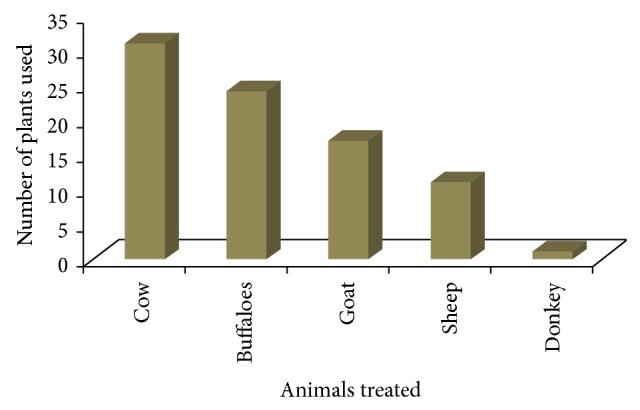
Number of plants used to treat different domestic animals.

**Figure 3:**
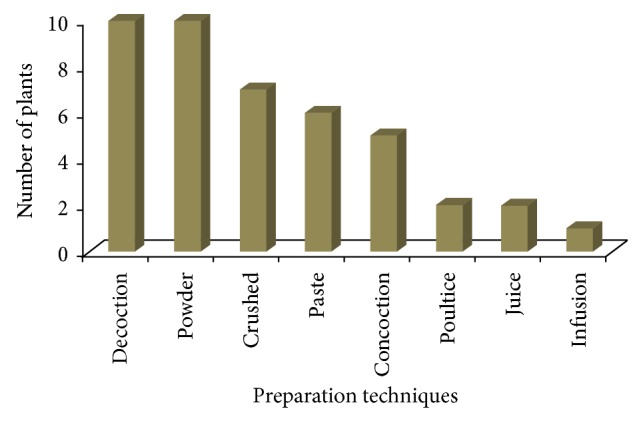
Traditional medicines preparation.

The present study revealed that local farmers of Kohat region utilize 41 medicinal plants belonging to 30 families for the treatment of different types of livestock ailments (Table 1). Among all the families, Asteraceae was found to be dominant (4 species) in the study area being in use in ethnoveterinary practices in the region. Traditional farmers mostly used herbs (55%) for the preparation of ethnomedicines (Table 2) followed by shrubs (27%). Almost all plant parts were being used for medicinal purposes but leaves (47%) were found to be the most frequently used plant part followed by whole plant (32%) and roots (17%) (Table 2). Local farmers used these ethnomedicines to treat different types of domestic animals like buffaloes, cows, goats, sheep, and donkeys. A total of 31 plants were found to be used for treatment of cow ailments followed by 24 plants against buffalo's ailments and 17 for goats (Figure 2). Different types of ailments were treated which were categorized into 8 major categories. Gastrointestinal infections were found to be most common in domestic animals and a total of 13 plants were used against them followed by 7 plants which are used as antipyretic while 6 are used for wounds treatment (Table 3). Local farmers prepare different types of ethnomedicines but the most preferred techniques were decoction and powder (10 plants each) followed by crushing (7 plants) in the studied region (Figure 3). Monotherapy was most common in the study area; only few plants were found to be used in concoction form (Table 1). For example, stem of* Allium sativum* is mixed with flower of* Punica granatum* and milk and used against gastrointestinal infection; roots of* Asparagus gracilis* are mixed with leaves of* Coriandrum sativum* to make fine concoction and given with water to cattle for delivery purposes. Different types of vehicles were found to be used for preparation and administration of plant recipes like sugar, flour, water, and milk (Table 1). The most common route of administration was oral (75%) followed by dermal (24%) and only single species is administered through nasal pathway (Table 1). Recovery time of majority of the recipes was three to four days. Informant consensus (Fic) results have shown a high degree of consensus for gastrointestinal, respiratory, and reproductive (0.95 each) ailments, which were followed by parasitic infections and wound healing (0.90 each) (Table 3). The highest plant use citation was for gastrointestinal (260) followed by wound healing (53) and reproductive (47) ailments. The present study revealed 10 medicinal plants having high FL value (Table 4). FL values in this study varied from 1.0% to 100%.* Asparagus gracilis* ranked first with the highest FL value (93%) followed by* Rumex hastatus* ranked second (91%),* Tinospora cordifolia* ranked third (90%), and* Aloe barbadensis* ranked fourth (85%). The entire informants interviewed were aged people ranging between 40 and 70 years old. No use of ethnoveterinary medicine by women or young generation was recorded.

## 4. Discussion

Livestock keeping is one of the most important economic sources of rural community of study area. The farmers and nomadic people of the area not only depend on plants to get fodder for their animals but also use different medicinal plants to treat various animal diseases. The majority of the people interviewed using ethnoveterinary plants have got this knowledge from their forefathers while some have learned from the other people. The majority of the farmers and nomadic pastoralists were not very well off and heavily dependent on medicinal plants due to their unaffordable potential of using modern veterinary drugs for their animals treatment.

The present study revealed that people of the region use 41 medicinal plants for their livestock health care. Similar studies have also been documented in other parts of Pakistan [5, 15]. Traditional healers of the region mostly use herbs for the treatment of their animals that might be due to the fact that herbs are available everywhere and easy to collect as compared with other growth forms. The results indicate the abundance of herbs in the study area and their high usage might also be due to the strong efficacy of herbaceous plants against livestock ailments. The same findings were also reported from other studies conducted at different parts of the world [16, 17].

The wider utilization of this Asteraceae family might be due to its higher abundance in the study area or might be due to high bioactivity. Similar studies have also been reported from other parts of world [18] and from Pakistan [19, 20] where traditional healers mostly use the member of Asteraceae family for the preparation of traditional medicines for the treatment of different livestock and human ailments. This observation is however different from that of Appidi et al. [21] and Offiah et al. [22] who in an ethnoveterinary survey reported Fabaceae family as the highest. The difference among studies might be related to the different dominant vegetation of the areas or might be associated with traditional beliefs of different cultures in using specific plants traditionally.

Most of the ethnoveterinary recipes in the study region are prepared using leaves of plants. The highest use of leaves in large number of ethnomedicinal and ethnoveterinary studies has also been documented from different parts of the world [23, 24]. Preferred use of leaves might be associated with ease of collection as compared to other plant parts. Leaves are the main site of photosynthetic apparatus and are involved in a variety of physiological processes of plants and produce numerous secondary metabolites that could be a possible reason for their effectiveness and efficacy against different livestock diseases. Local people also use whole plants after leaves for herbal formulation that could be a very destructive type of harvesting for rare and slowly growing plants from conservation point of view. Harvesting of leaves does not pose any serious impact on the life cycle of plants and is considered a sustainable type of harvesting. The present results are in contradiction with other studies where roots are the most widely used plant part in ethnoveterinary practices [25, 26].

Cows and buffaloes were the most commonly treated animals followed by goats and sheep in the studied region. Similar results have also been conducted by van der Merwe et al. [27] and Benítez et al. [16]. There was almost no mention of treating dogs, cats, or donkeys. This is probably because rural people do not generally keep animals as pets and because nonproduction animals are perceived as being more resistant than humans to different kinds of ailments. Production animals are also more important because of their socioeconomic importance in the local inhabitant life. The majority of the plants in the region are used to treat different types of gastrointestinal problems of the livestock like diarrhea, expulsion of worms, constipation, and so forth. It has already been found that stomach infections are more common in lactating animals which might be due to poor quality of fodder and drinking water [17]. Informant consensus results also showed the highest informant citation for gastrointestinal, respiratory, and gynecological problems. The highest informant citation against these infections gives an indication of high prevalence of these diseases in the region. According to Heinrich et al. [11], high Fic values are very useful in the selection of specific plants for further search of bioactive compounds. Widely used medicinal plants for species ailments always score the highest fidelity level. The present study determined different plants like* Asparagus gracilis, Rumex hastatus, Tinospora cordifolia, Aloe barbadensis*, and so forth, scored highest fidelity values and should be further subjected to phytochemical and pharmacological investigation to prove their medicinal efficacy.

The method of drug preparation in many cases varied from individual to individual. The same plant material for the same ailment was prepared in different ways by different traditional veterinary healers. Traditional healers prepare ethnoveterinary recipes mostly in the form of powder and decoction in the study area. Deeba [28] powdering or boiling is the most common method of drugs extraction. These findings are in line with a study conducted in the Malakand valley of Pakistan [5] while they are contradictory with the studies conducted in other parts of the world [29, 30]. Most of the recipes are prepared using single plant mixture while some of the recipes are also prepared in the form of concoction and it is generally believed that potency of the drugs can be enhanced when used in concoction form [31]. These recipes are mostly taken orally in the study area due to the high prevalence of the internal diseases. These recipes are given to the livestock with their feed along with different types of ingredients like sugar, flour, milk, and so forth, in the region. Similar findings are also reported from other regions of the world [18, 32]. The use of these vehicles might be due to their enhancing potential of taste and medicinal properties of certain plant remedies. Uniformity was lacking regarding amount of medicines to be used among informants during the interview. It was determined that a contradiction in ethnoveterinary dosage is a serious drawback of traditional medicinal plants. Informants only provided the knowledge of observed time of recovery of animals in response to given recipes. Full recovery is confirmed when the animals restart proper feeding and activities. Similar findings are also reported by other ethnoveterinary studies conducted elsewhere [5, 22].

It was confirmed from the present study that men had better knowledge regarding ethnoveterinary practices as compared to women. The reason might be due to the fact that men are mostly favored in shift of knowledge while women in the majority of the cultures are considered for family's care. This noticeable gender bias reflects the limited involvement of women in cattle production and herd health in Kohat region of Pakistan. Aged males had much more indigenous knowledge as compared to young generation which might be due to the lack of interest in such practices. Therefore, documentation of ethnoveterinary practices is an essential step toward the conservation of such knowledge before its extinction.

## 5. Conclusions

Local farmers and nomads of the region utilize different medicinal plants for the treatment of livestock due to their low income status and high expenses of western drugs. Traditional healers possess tremendous expertise in preparing herbal formulations of medicinal plants. Gastrointestinal infections were most common in the studied region; therefore attention should be given to provide good quality fodder and water to the livestock. Plants with high informant consensus and fidelity level should be subjected to further* in vitro* investigation for their phytochemical analysis and pharmacological activities. Young generation should be mobilized to take interest in ethnoveterinary practices in order to conserve this knowledge.

## Questionnaire of Ethnoveterinary Data Collection

*Informants' Consent for the Participation in the Study*

I__________ (name of informant) hereby give my full consent and conscious to participate in this study and declare that to the best of my knowledge the information that I have provided is true, accurate, and complete.

Date _____________ (signature/thumb impression of informant).

*Informants' Details*

 Name
 Gender
 Age
 Occupation
 Education
 Location/residence

*Data about Medicinal Plant and Its Use*

 Number of plants known
 Names of plants (local names)
 Plant part used
 Name of disease(s) treated
 Type of animal treated
 Method of crude drug preparation
 Use of single or mixture of plants
 Ingredients used
 Recovery time
 Route of administration
 Dosage

*Informant Consensus Factor and Fidelity Level*

 Name of plants used against disease category 1
 Name of plants used against disease category 2
 Name of plants used against disease category 3
 Name of plants used against disease category 4
 Name of plants used against disease category 5
 Name of plants used against disease category 6
 Name of plants used against disease category 7
 Name of plants used against disease category 8
 Name of plants used against disease category 9
 Name of plants used against disease category 10

*Remarks*

Plant identified as —— (botanical name and family)

Signature of researcher

## Figures and Tables

**Table 1:** Ethnoveterinary plants used for the treatment of livestock ailments in Kohat region.

Plant names/families name/voucher number	Local names	Habit	Part used	Ailment treated	Animal treated	Recipe	Vehicles	Route	Recovery
*Acacia modesta* Wall. Fabaceae KUH-421	Kikar	Tree	Leaves, seeds	Delivery	Buffaloes, cow	Decoction	Water	Oral	Two days

*Achyranthes aspera* Linn. Amaranthaceae KUH-422	Gishkay	Herb	Whole plant	Anthelmintic and delivery	Buffaloes, cow, goat, sheep	Powder	Sugar	Oral	One day

*Allium cepa* L. Amaryllidaceae KUH-423	Pyaz	Herb	Whole plant	Febrifuge, tonic	Sheep, goat	Decoction	Flour	Oral	Three days

*Allium sativum* L. Liliaceae KUH-424	Thoom	Herb	Stem	Gastrointestinal	Goat	Concoction	Milk	Oral	Ten days

*Aloe barbadensis* Mill. Liliaceae KUH-425	Kunwar	Shrub	Root	Gastrointestinal	Sheep, cow, buffaloes	Powder	Milk	Oral	Six days

*Artemisia brevifolia* Wall. Asteraceae KUH-426	Jaukay	Herb	Leaves	Removal of placenta	Cow	Decoction	Sugar	Oral	

*Asparagus gracilis* Royle. Liliaceae KUH-427	Lachgawa	Herb	Root	Delivery	Goat	Concoction	Water	Oral	

*Brassica campestris* L. Brassicaceae KUH-428	Sarson	Herb	Whole plant	External lice (blood feeding)	Cow, buffaloes	Paste		Dermal	Five days

*Calotropis procera* (Willd.) R.Br. Apocynaceae KUH-429	Spulmaey	Shrub	Fruit, leaves	Intestinal worms and skin infections	Buffaloes, cow, goat, sheep	Paste, concoction	Sugar	Oral, dermal	Three days

*Cannabis sativa* L. Cannabaceae KUH-430	Bhang	Shrub	Leaves	External parasite, appetizer	Cow, donkey, buffaloes	Poultice, powder		Dermal, oral	Two days

*Chenopodium album* L. Amaranthaceae KUH-431	Samaray	Herb	Whole plant	Wound healing	Goat, sheep, cow	Paste		Dermal	One day

*Chrysanthemum leucanthemum* L. Asteraceae KUH-432	Chitti pulari	Herb	Whole plant	Increase milk production	Goat, cow, buffaloes	Powder	Flour	Oral	Continuously

*Citrullus colocynthis* (L.) Schrad. Cucurbitaceae KUH-433	Karthuma	Herb	Root	Skin infection	Buffalo, cow, goat, sheep	Juice		Dermal	Two days

*Convolvulus arvensis* L. Convolvulaceae KUH-434	Shankpuspi	Herb	Whole plant	Constipation	Cow, buffaloes, sheep	Crushed	Sugar	Oral	Four days

*Coriandrum sativum* L. Apiaceae KUH-435	Dhania	Herb	Leaves, root	Antidiuretic	Buffaloes	Decoction	Water	Oral	One week

*Curcuma longa* L. Zingiberaceae KUH-436	Haldi	Tree	Leaves	Wound healing	Cow	Decoction	Sugar	Oral	Two days

*Cynodon dactylon* L. Poaceae KUH-437	Wakha	Herb	Whole plant	Wound healing, analgesic	Cow, buffaloes	Concoction	Milk	Oral	Two days

*Cynoglossum lanceolatum* Forssk. Boraginaceae KUH-438	Pachy	Herb	Root	Common cold	Cow, buffaloes	Crushed	Water	Oral	Two days

*Cyperus niveus* Retz. Cyperaceae KUH-439	Kulio	Herb	Whole plant	Common cold, stomach worms, joint pains	Cow, goat	Crushed	Water	Oral	Four to five days

*Datura inoxia* Mill. Solanaceae KUH-440	Mangaz	Herb	Whole plant	Antilice	Sheep, cow	Paste	Milk	Dermal	Three days

*Hedera nepalensis* K. Koch Araliaceae KUH-441	Zalai	Shrub	Leaves	To remove leeches	Sheep	Infusion		Nasal	Three days

*Melia azedarach* L. Meliaceae KUH-442	Dhrek	Tree	Leaves	Stomach flatulence	Cow, buffaloes	Powder	Sugar	Oral	Four days

*Mentha arvensis* Linn. Lamiaceae KUH-443	Pudina	Herb	Leaves	External parasite	Cow	Paste		Dermal	Four days

*Morus alba* L., Moraceae KUH-444	Toot	Tree	Leaves, fruit	Laxative	Buffaloes	Crushed	Milk	Oral	Two days

*Morus nigra* L. Moraceae KUH-445	Tor toot	Tree	Leaves	Tonic, laxative	Cow, buffaloes	Powder	Water	Oral	Three days

*Nerium oleander* L. Apocynaceae KUH-446	Ghanderay	Shrub	Whole plant	Stomachache	Sheep	Concoction	Water	Oral	One week

*Ocimum basilicum* L. Lamiaceae KUH-447	Kashmalay	Shrub	Leaves	Gastrointestinal	Buffaloes	Decoction	Water	Oral	Four days

*Punica granatum* L. Lythraceae KUH-448	Anar	Shrub	Fruit, leaves	Anthelmintic	Cow, buffaloes, goat	Decoction	Milk	Oral	Two days

*Ricinus communis* Linn. Euphorbiaceae KUH-449	Arund	Shrub	Leaves, stem	Common cold	Buffaloes, cow	Powder	Flour	Oral	Three days

*Rumex hastatus* D. Don. Polygonaceae KUH-450	Tarooky	Shrub	Root and leaves	Wound healing	Goat, cow, buffaloes	Powder	Flour	Oral	Four days

*Solanum surrattense* Burm. f. Solanaceae KUH-451	Kandiari	Herb	Whole plant	Fever, cough, intestinal infections	Cow, buffaloes, goat, sheep	Crushed	Flour	Oral	One week

*Sonchus asper* (L.) Hill. Asteraceae KUH-452	Spingul	Herb	Whole plant	Milk production	Goat, cow, buffaloes	Decoction	Flour	Oral	Continuously

*Tagetes minuta* L. Asteraceae KUH-453	Ban hanjari	Herb	Leaves	Skin infections	Cow, buffaloes	Juice		Dermal	Five days

*Tamarix aphylla* (L.) H. Karst. Tamaricaceae KUH-454	Khagal	Tree	Leaves	Kill worms of wounds	Cow	Paste		Dermal	Four days

*Tinospora cordifolia* Miers. Menispermaceae KUH-455	Giloe	Climber	Whole plant	Skin infections	Cow, goat	Poultice		Dermal	Three days

*Tribulus terrestris* Linn. Zygophyllaceae KUH-456	Markondai	Herb	Whole plant	Chronic cough	Cow, buffaloes, goat	Crushed	Sugar	Oral	One week

*Trifolium repens* L. Papilionaceae KUH-457	Shoutal	Shrub	Root	Tonic, laxative	Goat, cow	Powder	Flour	Oral	One day

*Triticum aestivum* L. Poaceae KUH-458	Gandam	Herb	Seeds	Common cold, dysentery	Cow	Powder	Flour	Oral	Two days

*Verbena officinalis* L. Verbenaceae KUH-459	Shamakay	Herb	Stem, leaves	Wound healing	Buffaloes	Decoction	Milk	Oral	Three days

*Vitex negundo* L. Verbenaceae KUH-460	Marmandi	Shrub	Stem	Mange, fever, stomach problems	Cow, goat	Crushed, decoction	Sugar	Oral	Four to six days

*Zizyphus nummularia* W. & A. Rhamnaceae KUH-461	Kurkunda	Tree	Leaves	Wound healing	Cow	Decoction	Sugar	Oral	Three days

**Table 2:** Habit and parts used of ethnoveterinary plants.

General attributes	Total plants	Percentage
Habit		
Herbs	22	55
Shrubs	11	27
Trees	06	15
Climbers	01	02
Parts used		
Leaves	19	47
Whole plant	13	32
Root	07	17
Stem	04	10
Fruit	03	07
Seed	01	02

**Table 3:** Informant consensus factor.

Disease categories	Nur	Nt	Fic
Gastrointestinal	260	13	0.95
Respiratory	22	02	0.95
Reproductive	47	03	0.95
Dermatological	12	04	0.77
Wounds	53	06	0.90
Antipyretic	14	07	0.53
Parasitic	45	05	0.90
General body tonic	09	03	0.75

**Table 4:** Fidelity level of highly utilized species.

Number	Plant species	Disease category	Ip	Iu	FL %
01	*Asparagus gracilis *	Reproductive	29	31	93
02	*Rumex hastatus *	Wound healing	32	35	91
03	*Tinospora cordifolia *	Dermatological	28	31	90
04	*Aloe barbadensis *	Gastrointestinal	23	27	85
05	*Convolvulus arvensis *	Gastrointestinal	24	29	82
06	*Tribulus terrestris *	Respiratory	17	21	80
07	*Zizypus nummularia *	Wound healing	18	23	78
08	*Chenopodium album *	Wound healing	17	25	68
09	*Artemisia brevifolia *	Reproductive	19	30	63
10	*Cannabis sativa *	Parasitic	17	29	58
